# The practical and ethical challenges of identifying, accessing and obtaining school canteen transactional data for public health research

**DOI:** 10.1177/20552076241297356

**Published:** 2024-12-05

**Authors:** Alice Gilmour, Ruth Fairchild

**Affiliations:** 1 School of Sport and Health Sciences, Cardiff Metropolitan University, Cardiff, UK

**Keywords:** Adolescents, school food, big data, sales data, health research, public health, food choice, purchases, health behaviour

## Abstract

**Objective:**

The purpose of the study was to investigate the feasibility of using existing data to better understand what pupils purchase during the school day and its nutritional quality. This report highlights the ethical challenges experienced in attempting to obtain anonymised school canteen transaction data for public health research.

**Methods:**

Semi-structured interviews were conducted before a variety of approaches were tried to recruit secondary schools for the study via purposive sampling.

**Results:**

Barriers encountered included (i) identifying data providers, (ii) identifying data owners, (iii) data sharing and (iv) engaging stakeholders. The approaches taken to mitigate each of these barriers and subsequent ethical issues are summarised so that future researchers are aware of any potential problems. Failure to overcome these blockages meant that the original study had to be curtailed.

**Conclusions:**

School canteen transactional big data remains an underutilised research resource which has immense potential for understanding adolescents’ dietary choices and nutritional intake across the school day. The inaccessibility of anonymised datasets for public health research and complex political issues surrounding data ownership and sharing must be discussed and effort should be made to circumvent barriers. Establishing the current landscape of school food and drink would be beneficial for policymakers, educators and public health researchers.

## Introduction

There is increasing pressure upon public health agencies to use data to drive better decision-making and inform policy.^1^ Although School Food Standards (SFS) are in place across the United Kingdom (UK) and a large proportion of daily food and drink intake occurs during the school day,^2,3^ little is known about what pupils choose to consume whilst at school.^4^ Nevertheless, individual-level canteen purchase data is being collected across the majority of UK secondary schools via cashless payment systems. Payments via contactless cards, biometric thumb or fingerprint scanning reduces peer identification and stigmatisation of pupils in receipt of free school meals.^5^

Although collection of individual-level transaction data is widespread; this big data is seemingly unused by either the school, local authority (LA) or government and its scrutiny by catering providers and cashless catering system (CCS) providers remains unknown. There is great public health research potential in these datasets, but utilising school canteen transactional data remains a novel data collection method that has been little used.^4,6,7,8^

Beneficially, transactional data is continually compiled unobtrusively, involving no participants nor subsequent participatory effects. Exploiting already situated CCS technology to collect current or retrospective individual-level (i.e., year group, gender) big data from school canteen transactions has immense potential for ministries of health and education.^7^ The longitudinal assessment of food choice behaviour serves as a baseline from which health policy can be planned, as well as inform healthy eating interventions and innovation.^9^

The aim of the study was initially to investigate how feasible it is to use the data from secondary school canteen cashless payment systems to understand what food and drink are purchased at school and its nutritional quality. The UK General Data Protection Regulation (GDPR) is a data privacy and security law for personal data, regulating protection, accountability and consent. Fully anonymised data does not fall under UK GDPR.^10^ Moreover, assessment of public interest (i.e., whether the data should be shared) falls to the data controllers, defined as the individual or organisation which determines the purpose and processing of personal data.^10,11^

This report summarises the practical and ethical issues encountered in trying to obtain secondary school canteen transaction data from 2022 to 2024 in a constituent part of the UK so that future researchers are aware of possible challenges. Unsurmountable obstacles meant that only the first phase of the planned methodological approach was completed (see Box 1).

Box 1. Planned methodology for the study.

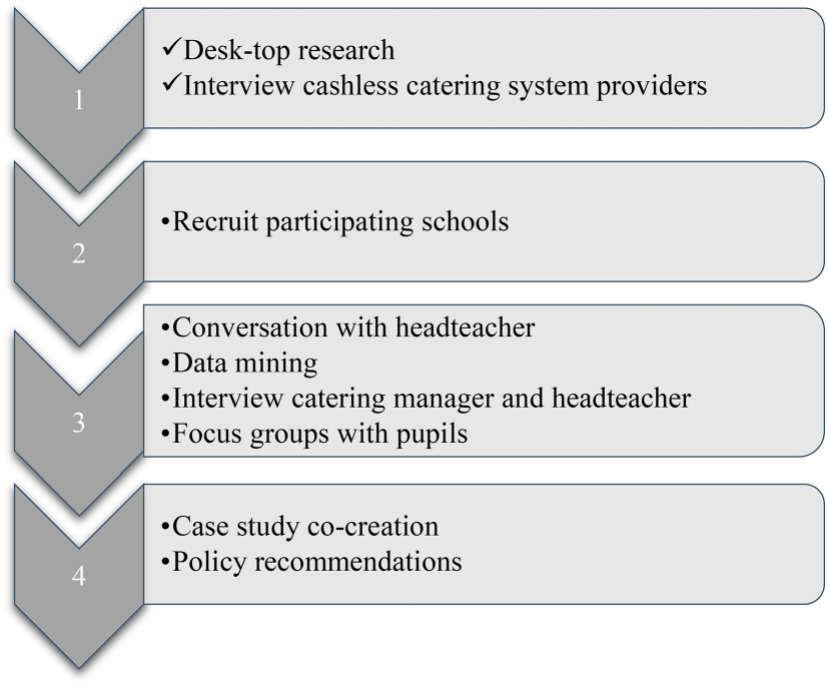

## Identifying CCS providers

First, identifying which CCS providers are in use across secondary schools via desk-top research proved challenging as this information was not publicly available. Rather, individual school and LA webpages link to the cashless payment system apps and websites. However, through contacts at the national representative organisation of LAs (NROLA), all seven CCS providers were identified. A representative from each CCS provider was emailed information about the study and provided informed consent before being interviewed over Microsoft Teams. These semi-structured interviews sought to explore how the cashless catering software functions, integrates and its capabilities concerning school food transactions. Interviews were audio recorded and transcribed before being thematically analysed using NVivo QSR 12; the key themes identified were: (i) the benefits of cashless catering; (ii) issues and capabilities of the data; and (iii) the need to integrate with other software. It was found that although cashless payment systems and CCS work in tandem, the catering management software is on the periphery and a secondary calculation is required to calculate the nutritional content of food and drink sold based on transaction data. In hindsight, data ownership^1^ should have been discussed before the recruitment phase.

## Identifying data owners

Prior school canteen transactional data studies sought permission from the head teacher,^8^ so accordingly, the researchers had supposed that schools were the data owners and the head teacher had authority over datasets. Thus, ethical documentation (i.e., information sheet, informed consent form and questioning schedules) were developed for secondary school head teachers in preparation for phase three of the planned methodology. Triangulation of four methodological approaches would have provided insight into how software functions, calculates nutritional values of food, whether extracted data is a reliable indicator of consumption and to establish current use of systems plus any barriers preventing maximum utilisation in the school setting.^1^

Recruitment took place via convenience sampling, utilising valuable existing relationships between the public health agency and Healthy School Coordinators.^2^ Initially, the aim was to carry out the study with nine schools. However, only five schools responded to the convenience sampling and gave permission for the research team to contact them via email (see Box 2). Identifying whom owns the data became an apparent issue and stalled any access to the data. Despite the NROLA stating that LAs owned and controlled the data, School 5 discovered that they were the data controller.^2^

Box 2. Decisions on whether anonymised secondary school canteen transaction data could be shared for public health research purposes.

*School 1* – The headteacher postponed study commencement for several months as they continued to suggest later dates that they would be able to begin. Eventually, they declined any participation as they were too busy and at ‘capacity’. (Time to decision: 6 months.)

*School 2* – The school contact was keen to participate but cited two barriers. First, an existing data sharing agreement with the government; and second, their contracted catering company declared that the data was too commercially sensitive to disclose. (Time to decision: 2 days.)

*School 3* – This school did not respond to five emails and four follow-up phone calls. Liaison from a Healthy School Coordinator led to the school’s headteacher being willing to participate. However, after numerous failed meeting attempts, the headteacher declined any participation due to lack of ‘capacity’. (Time to decision: 6 months.)

*School 4* – The assistant headteacher met with the researcher but later had to decline any further participation in the study. The reason given was because the catering was LA-organised rather than school-organised and the LA did not permit data sharing. (Time to decision: 3 weeks.)

*School 5* – Again, liaison with a Healthy School Coordinator resulted in this school being engaged and willing to participate. The school contact met with the researcher as well as their CCS provider alongside a LA Data Protection Officer (rather than LA caterers) and discovered that the school is the data controller. The school contact phoned the CCS provider to request data access. Despite a promising start, the school failed to respond to numerous emails and phone calls to provide the data. (Time to decision: 4 months.)

## Data sharing

As school canteen transactional data can be extracted in an anonymised format,^4,6,8^ it does not fall under the UK GDPR.^10^ Further, if schools own and control their pupils’ data, the sharing of anonymised data for public health benefit is deemed acceptable.^11^ Yet, due to data sharing agreements between the CCS providers and LA or school, the seven providers interviewed were unable to disclose which areas they operated in. This may have allowed the researchers to take a more purposive sampling approach, contacting specific schools for consent if they had a provider who was also willing to share the transaction data, mitigating the hurdle of obtaining data-sharing permissions from the school.^12^ Nowadays, school caterers are under increasing pressure to provide healthy school meals within ever-tightening budgets during the cost-of-living crisis.^3^ Understandably, these societal factors possibly contributed to the vulnerability and scepticism felt around any potential misuse and scrutiny of big data.^13^

From the outset, it was made clear to all stakeholders that the data would only be used in an anonymised format and the purpose of the study was to simply establish whether it was feasible to obtain and utilise transaction data to understand what pupils purchase. It was not to measure compliance to the existing SFS. The lack of official guidelines on data sharing^14^ between schools, LAs and the NROLA slowed and eventually stalled school recruitment.

## Engaging stakeholders

A range of stakeholders were engaged in the recruitment phase (see Box 3). It was difficult to engage busy school stakeholders in the study. Extant literature widely acknowledges that recruiting schools and school staff to participate in research can be a struggle as busy schedules and time pressures make recruitment difficult.^15^ To mitigate this, liaison with the Healthy School Coordinators meant that only schools willing to cooperate in the study were initially contacted.^16^ The researchers acknowledged that LAs and schools worried that the study could draw attention to their SFS compliance – or lack thereof – and were unwilling to take that risk. Despite clear communication outputs (i.e., information sheets) from the research team, the NROLA was apprehensive in allowing the project to proceed. Notwithstanding, power imbalances between policy and the school food environment^3^ remain a long-standing ‘emotive political and social issue’.^6^^, p. 209^

Box 3. Stakeholders to consider in similar studies. LA contacts could include:

Healthy school coordinators

Lead for health-promoting schools

Data protection officer

School contacts could include:

Headteacher

Assistant headteacher

Well-being lead

Leader of learning

Catering manager

## Discussion

The researchers experienced concurrent ethical and practical challenges with identifying data providers, data owners, data sharing and stakeholder engagement. The practical and ethical issues accessing and obtaining school canteen transactional data for public health research were unanticipated.

Comparisons can be made between school transaction data and retailer loyalty card data. Both are routinely collected and accumulate continuously over time.^17^ There is an ever-growing pool of studies utilising loyalty card data to understand lifestyle choices and consumption patterns.^13^ Similar to school canteen transaction data, the existing data infrastructure is already in place yet remains an underutilised resource although a greater convergence of datasets is becoming increasingly possible.^17^ Additionally, prevailing attitudes towards sharing loyalty card data are rational and individuals are willing to donate their data for public health research.^18,19^ Progress in loyalty card data research should be easily transferrable to school canteen transaction data if only the practical and ethical issues raised in this report can be ameliorated. Comprehending today's food landscape is dependent on systematic, meaningful linkages between food transactions (i.e., barcode data), dietary measures and nutritional information.^13,20^

The failure to access school canteen transaction datasets in this study indicated an unwillingness or refusal to share data. This is arguably not in the best interests of the public. Studies show that the general public approve of administrative data linking for research projects as long as data is anonymised, kept secure and there is social value.^11,19^ The transaction data would have served as a baseline, so that ministries of health and education could comprehend what is eaten at present before planning school health policy. Perhaps instead of taking a public health approach to this study, the researchers would have had better success exploring the commercial nature of datasets for school meal providers.

In retrospect, the NROLA could have been engaged initially but was not as it had been assumed that schools would provide permission for data access via the CCS providers. An alternative approach could be to access dietary data via individual school children and parental consent as it is known that parents have access to their child's canteen transactional data online.^21^ Unfortunately, this would be very time-consuming at a national level.

Notably, there were three limitations associated with this study: (i) it was conducted in one constituent part of the UK, so extrapolation of findings may lack relevancy; (ii) time constraints meant that recruitment approaches could not continue; and, (iii) a lack of engagement in the recruitment and thereafter. Overall, public health data sharing can be challenging, but change is needed and data-sharing policies must be developed to allow this.^1,22^ School CCS data has been collected successfully elsewhere in the UK with no data-sharing issues.^4,6,7,8^

## Conclusion

In-situ CCSs are a significant source of data for providing immense insight into the nutritional quality of food served across secondary schools. This report demonstrates the multifaceted and complex ethical issues derived from trying to access transaction data for public health research.

## Supplemental Material

sj-docx-1-dhj-10.1177_20552076241297356 - Supplemental material for The practical and ethical challenges of identifying, accessing and obtaining school canteen transactional data for public health research Supplemental material, sj-docx-1-dhj-10.1177_20552076241297356 for The practical and ethical challenges of identifying, accessing and obtaining school canteen transactional data for public health research by Alice Gilmour and Ruth Fairchild in DIGITAL HEALTH

## Notes

1. Data owner: Similar to ‘data controller,’ in that they have authoritative rights over the data.^10^

2. Local support of health and well-being in schools.
