# Supplementary material for: The practical and ethical challenges of identifying, accessing and obtaining school canteen transactional data for public health research
Source: Digit Health. 2024 Dec 5;10:20552076241297356. doi: 10.1177/20552076241297356 (PMC11622285; doi:10.1177/20552076241297356)
Supplement: sj-docx-1-dhj-10.1177_20552076241297356 - Supplemental material for The practical and ethical challenges of identifying, accessing and obtaining school canteen transactional data for public health research [file sj-docx-1-dhj-10.1177_20552076241297356.docx]

Interview Questions

COMPANY:

1. How many schools do you operate in? UK-wide? In this constituent part of the UK?
2. How many updates or versions are currently being used by schools? What is the difference between the various versions?
3. Proportionately, do you know how many schools use cards versus biometrics? Are there any other methods pupils use to pay?

USERS:

1. Can schools individually purchase the cashless system, or do Local Authorities tend to purchase for schools in the area? How would a school go about procuring a system?
2. Is training provided for new users? For school staff? For parents? Do you, or the school take responsibility for assisting parents with app/website usage?
3. Do schools/pupils/parents generally seem keen to use a cashless system, or is there evident reluctance to change? Do individuals need convincing/reassuring that data will be scored securely? *(i.e. an invasion of privacy?)*
4. Have some schools switched from another cashless system provider, or do you tend to be the first system they have used?
5. Has there been an increase in the use of cashless systems since the pandemic began? *(Perhaps using the system for other communications or payments, not only school meals.)*
6. Can the sales database be downloaded into an Excel or SPSS file, or may it only be viewed through the system? How does this work? i.e. do schools receive a daily/weekly dataset of all food items purchased school-wide? Is this anonymised? How might a school use this data? *Is there the facility for parents to request that the school prints out a record of what their child has been purchasing at school?*

KITCHEN:

1. Can the cashless system be linked to a nutrition analysis software, or do catering staff use another software? Is this optional? What nutritional analysis software do users of your software tend to use?
2. Is the cashless system linked to stock? (Does this enable catering managers to calculate food waste?)
